# Supplementary material for: Lessons from implementation research on community management of Possible Serious Bacterial Infection (PSBI) in young infants (0-59 days), when the referral is not feasible in Palwal district of Haryana, India
Source: PLoS One. 2021 Jul 7;16(7):e0252700. doi: 10.1371/journal.pone.0252700 (PMC8279773; doi:10.1371/journal.pone.0252700)
Supplement: S1 Checklist — (DOCX) [file pone.0252700.s001.docx]

# **Observation Tool 1:**

# **(Observation of Medical Officers on Assessment of Sick Young Infants )**

*Note: The present tool is developed to observe how medical officers assess and manage sick young infants at various health facility*

## **Section IA – Basic Information**

| **Q.No** | **Particular** | | **Response** |
| --- | --- | --- | --- |
| **Session general Information** | | | |
|  | observation made at | | CHC……………………….……………..…...1  PHC…………………………………………..2  sc…………………..…………….….……..3 |
|  | Name of the Facility | |  |
|  | Date of clinic ( DD/MM/YY) | | / / |
|  | Timing of the ANC clinic | Start | :  AM/PM |
|  |  | End | :  AM/PM |
| **Observer Information** | | | |
|  | Name of the observer: | |  |
|  | Designation: | |  |
|  | Timing for observation | Start ( HH/MM) | :  AM/PM |
|  |  | End (HH/MM) | :  AM/PM |
| **Observation of routine Check up** | | | |
| ANC जांच के दौरान दी गयी सेवाओं का ऑब्जरवेशन | | | |
|  | Age of the infant | | ( In days) |
|  | Complaints by Mother/ Caregiver while presenting sick young infant in the clinic ( Descriptive) | | |
|  | Did the medical officer asked “ how is the infant doing” (Asked about general health condition) | | Yes…..……………………………1  No…….…………..………..……..2 |
| **Danger Sign Assessment ( Did the MO assessed followings)** | | | |
|  | Unable to take feed or not feeding well or not feeding since birth | | Yes…..……………………………1  No…….…………..………..……..2 |
|  | Not moving or movement when stimulated | | Yes…..……………………………1  No…….…………..………..……..2 |
|  | Convulsion | | Yes…..……………………………1  No…….…………..………..……..2 |
|  | Fast breathing (RR 60 or more) | | Yes…..……………………………1  No…….…………..………..……..2 |
|  | Severe Chest In-drawing | | Yes…..……………………………1  No…….…………..………..……..2 |
|  | Fever (or feels hot to touch) | | Yes…..……………………………1  No…….…………..………..……..2 |
|  | Hypothermia (or feels cold to touch) | | Yes…..……………………………1  No…….…………..………..……..2 |
|  | Assessed completely for other sickness danger signs | | Yes…..……………………………1  No…….…………..………..……..2 |
|  | IMNCI Recording form used while assessment | | Yes…..……………………………1  No…….…………..………..……..2 |
| **What actions taken by Medical Office/assessor** | | | |
|  | Classification sickness made | | Yes…..……………………………1  No…….…………..………..…….2 (Skip to 22) |
|  | What was the classification | | PSBI............................................................1  Fast Breathing only...............................2  Other sickness.........................................3  No Classification....................................4 |
|  | Did the medical officer advised the family for a higher centre referral | | Yes…..……………………………1  No…….…………..………..…..….2  Not applicable…………………….3 |
|  | Did the medical officef precribed Inj Gentamicin | | Yes…..……………………………1  No…….…………..………...…….2  Not applicable ………..…..……..3 |
|  | Confidence of the doctor in handling sick young infant ( Qualitative) | | Yes…..……………………………1  No…….…………..………..…….2 |
|  | Called anyone else to discuss about the infant’s illness | | Yes…..……………………………1  No…….…………..………..…….2 |
