# Supplementary material for: Lessons from implementation research on community management of Possible Serious Bacterial Infection (PSBI) in young infants (0-59 days), when the referral is not feasible in Palwal district of Haryana, India
Source: PLoS One. 2021 Jul 7;16(7):e0252700. doi: 10.1371/journal.pone.0252700 (PMC8279773; doi:10.1371/journal.pone.0252700)
Supplement: S1 Fig — (DOCX) [file pone.0252700.s002.docx]

**
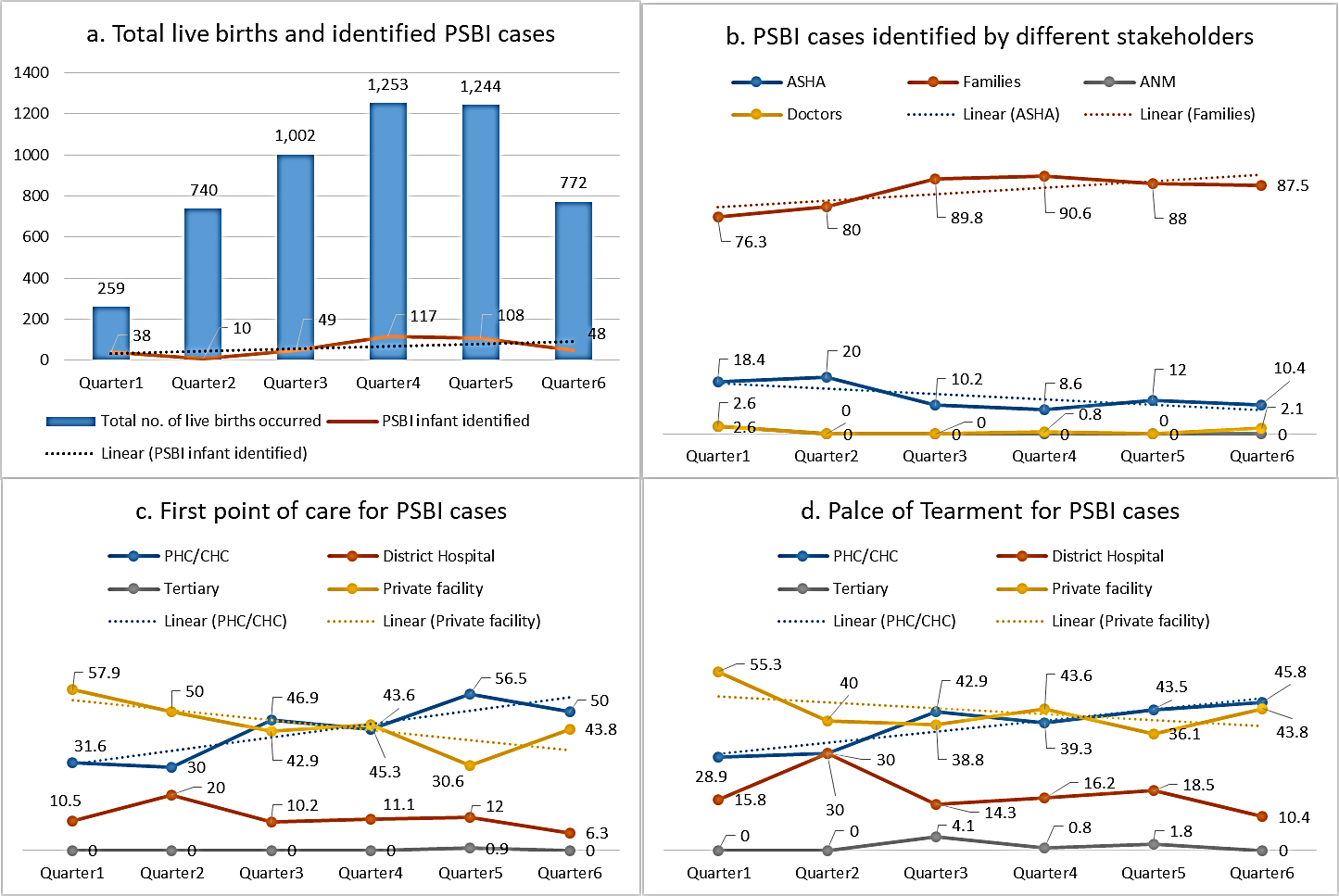
S1 Fig**. **Identification, first point of care, place of treatment and number of deaths of in young infants with PSBI signs across six study quarters (N- 370)**
