# Supplementary material for: Lessons from implementation research on community management of Possible Serious Bacterial Infection (PSBI) in young infants (0-59 days), when the referral is not feasible in Palwal district of Haryana, India
Source: PLoS One. 2021 Jul 7;16(7):e0252700. doi: 10.1371/journal.pone.0252700 (PMC8279773; doi:10.1371/journal.pone.0252700)
Supplement: S1 Table — (DOCX) [file pone.0252700.s003.docx]

| **S. no** | **Name of Private Facility** | **Number** |
| --- | --- | --- |
|  | AMINA HOSPITAL | 1 |
|  | APEX HOSPITAL | 1 |
|  | BALLABHGARH PVT HOSPITAL | 1 |
|  | BAMNIKHEDA CLINIC | 2 |
|  | BANIKRENT FB KALIKA HOSPITAL | 1 |
|  | BANSAL HOSPITAL | 3 |
|  | KAJAL NURSING HOME | 1 |
|  | KISHAN SINGH HOSPITAL HODAL | 1 |
|  | LIFE CARE HOSPITAL | 1 |
|  | HOSPITAL ANIL BAMS | 1 |
|  | MOOLCHAND HOSPITAL PALWAL | 4 |
|  | NIDAN HOSPITAL PALWAL | 5 |
|  | SACHIN HOSPITAL PALWAL | 7 |
|  | SANJEEVANI HOSPITAL BAMNIKHEDA | 3 |
|  | SATYA SAI HOSPITAL PALWAL | 1 |
|  | SEHRAWAT HOSPITAL PALWAL | 24 |
|  | SOMAARTH CLINIC | 35 |
|  | SHANTI MALIK HOSPITAL | 5 |
|  | VRIJ RAHUL HOSPITAL | 1 |
|  | WADHA HOSPITAL BAMNIKHERA | 1 |
|  | RAHUL CLINIC HODAL | 7 |
|  | RAHUL HOSPITAL, PALWAL | 5 |
|  | PRABHU DAYAL HOSPITAL | 2 |
|  | PVT. HOSPITAL SIKRI | 1 |
|  | P.V.T CLINIC MITROL | 1 |
|  | PALWAL CLINIC | 1 |
|  | DHARAMVEER CLINIC | 1 |
|  | PVT BAMNIKHERA | 2 |
|  | PVT CLINIC BHANGURI | 1 |
|  | PVT HODAL | 1 |
|  | PVT. CLINIC HATHIN | 1 |
|  | ROY CLINIC | 1 |
|  | PVT CLINIC | 4 |
|  | Name Not available | 2 |
|  | Informal health providers | 26 |
|  | **Total** | **155** |

**S1 Table. List of private providers who managed 155 PSBI infants during implementation research at district Palwal (Haryana, India)**
