# Supplementary material for: Lessons from implementation research on community management of Possible Serious Bacterial Infection (PSBI) in young infants (0-59 days), when the referral is not feasible in Palwal district of Haryana, India
Source: PLoS One. 2021 Jul 7;16(7):e0252700. doi: 10.1371/journal.pone.0252700 (PMC8279773; doi:10.1371/journal.pone.0252700)
Supplement: S2 Table — (DOCX) [file pone.0252700.s004.docx]

**S2 Table. Total Sickness Identified in 0-59 Days Old Infants during Implementation Period (Aug 2017 - Jan 2019)**

| **Parameters** | **N (%)** |
| --- | --- |
| 1. Total no. of births occurred | 5,270 |
| 1. PSBI infants including fast breathing only(7-59 days) | 370(7.0) |
| 1. Infants with other sickness | 296(5.6) |
| 3.1. Local infection* | 126(42.6) |
| 3.2. Jaundice | 47(15.9) |
| 3.3. Diarrhea | 109(36.8) |
| 3.4. Very Low Weight/Low Weight | 13(4.4) |

*Skin infection, umbilical cord redness, oral thrush (presented as feeding difficulty, n - 1)
